# In Search of Spinal Muscular Atrophy Disease Modifiers

**DOI:** 10.3390/ijms252011210

**Published:** 2024-10-18

**Authors:** Daria Chudakova, Ludmila Kuzenkova, Andrey Fisenko, Kirill Savostyanov

**Affiliations:** National Medical Research Center of Children’s Health of the Ministry of Health of the Russian Federation, 119991 Moscow, Russia

**Keywords:** spinal muscular atrophy, disease modifiers, *SMN1*, *SMN2*, hereditary autosomal recessive disease, neurodegeneration

## Abstract

The 5q Spinal Muscular Atrophy (SMA) is a hereditary autosomal recessive disease caused by defects in the survival motor neuron (*SMN1*) gene encoding survival motor neuron (SMN) protein. Currently, it is the leading cause of infantile mortality worldwide. SMA is a progressive neurodegenerative disease with “continuum of clinical severity”, which can be modulated by genetic and epigenetic factors known as disease modifiers (DMs). Individuals (even siblings) with the same defects in *SMN1* gene might have strikingly different types of SMA, supposedly due to the impact of DMs. There are several therapeutic options for SMA, all of them focusing on the restoration of the SMN protein levels to normal. Determining DMs and the pathways in which they are involved might aid in enhancing existing curative approaches. Furthermore, DMs might become novel therapeutic targets or prognostic biomarkers of the disease. This narrative review provides a brief overview of the genetics and pathobiology of SMA, and its bona fide modifiers. We describe novel, emerging DMs, approaches and tools used to identify them, as well as their potential mechanisms of action and impact on disease severity. We also propose several disease-modifying molecular mechanisms which could provide a partial explanation of the staggering variability of SMA phenotypes.

## 1. Introduction

The 5q Spinal Muscular Atrophy (SMA) is a rare (orphan) hereditary autosomal recessive neurodegenerative disease. Despite being an orphan disease, SMA is the leading genetic cause of infantile mortality, and the second most common among hereditary autosomal recessive disorders, outnumbered only by cystic fibrosis [1].

It is a progressive and debilitating condition with a “continuum of clinical severity” [2]. The current classification of SMA, formulated in 1992, is based on clinical severity and the age of onset [3]. There are five forms of SMA (types 0–4), type 0 being the most severe form of SMA and type 4 the least severe. Type 0 SMA is characterized by prenatal onset resulting in respiratory failure at birth; patients with type 1 SMA (Werdnig-Hoffmann disease, Online Inheritance in Man ID (OMIM) #253500) experience onset at 0–6 months, while being unable to sit; individuals with type 2 SMA (OMIM: #253550; Dubowitz disease) experience the onset before 18 months of age, when they can sit; type 3 (Kugelberg-Welander disease, OMIM #253400) is characterized by an onset after 18 months of age, when patients can stand or are ambulatory; and individuals with type 4 SMA (OMIM #271150) experience onset from adolescence to adulthood, when they are ambulatory [4]. Type 1 is the most common form, representing ~45% of total SMA cases, type 2 represents ~20% cases, type 3~30%, and type 4 less than 4% [5].

The so-called “classic” SMA, accounting to ~94–96% of cases, is caused by large deletions within the survival motor neuron 1 gene (*SMN1*, HGNC:11117, OMIM:*600354) or by a gene conversion from *SMN1* to its paralogue, survival motor neuron 2 gene (*SMN2, HGNC:11118,* OMIM *601627) [6]. There is a copy number polymorphism (CNP, variability of the total number of copies of functional genes between individual genomes) of both genes, and a tendency for patients with a higher number of *SMN2* copies to develop milder forms of SMA; for example, patients with type 1 SMA usually have no more than two copies of *SMN2* [7], whereas patients with type 2 often have three copies of *SMN2* [8], and so on and so forth. However, the determination of SMA severity is not as simple as this facile model implies, especially in the case of SMA Type 2 and Type 3. Indeed, patients with three *SMN2* copies can have SMA ranging from Type 1 to Type 3, and it seems that the number of *SMN2* copies alone cannot reliably predict the severity of SMA in such a case. The most trivial explanation of this phenomenon is the possibility that the *SMN2* gene might have modifying variants in its sequence, including partial deletions, which result in different levels of full-length SMN. Thus, the cells with the same copy number of the *SMN2* gene, with and without sequence alteration, would be phenotypically different. However, this is not always the case, suggesting other contributors stagger the variability of clinical presentation of SMA.

It is known that phenotypic manifestation of diseases that follow patterns of Mendelian inheritance (Mendelian diseases) can be modulated by several genetic, epigenetic, and other factors known as disease modifiers (DMs) ([9,10]). As a result, even individuals with the same causative DNA sequence variants can have very different phenotypes and clinical manifestations of a disease. This complicates diagnostics and prognostics and poses a challenge when selecting the optimal therapeutic regimen. On the other hand, identifying DMs for particular diseases helps to deepen the understanding of its pathogenesis and develop novel curative approaches.

There is a well-known phenomenon of the existence of unaffected individuals with homozygous deletions within the *SMN1* gene ([11] and others). Several so-called discordant SMA families have been described so far, with siblings having the same homozygous defects within the *SMN1* gene, but strikingly different phenotypes, including the absence of SMA symptoms, supposedly due to the impact of DMs. There are therapeutic options for SMA, all of them focusing on the restoration of the SMN protein levels to normal. DMs causing the aforementioned discordance of symptoms might become novel therapeutic targets or prognostic biomarkers of the disease, aid in enhancing existing curative approaches, and serve as tools to broaden the current knowledge of SMA pathogenesis.

The key findings on SMA modifiers have been summarized in several relatively recent comprehensive reviews ([12,13,14,15], to name but a few), once again highlighting the importance of the subject. However, since their publication, many works have broadened our knowledge on the subject; they will be discussed in this review in detail, alongside previously reviewed ones. This narrative review provides a brief overview of genetics and pathobiology of SMA, and its bona fide modifiers. We also propose (yet to be tested) hypotheses of possible molecular mechanisms underpinning SMA phenotype variability. Further, we describe novel, emerging DMs, approaches and tools used to identify them, as well as their potential mechanisms of action and impact on disease severity. This review focuses mostly on biology and pathogenesis of SMA in humans; however, we acknowledge the existence of immense amounts of data on SMA generated in animal models.

## 2. *SMN1* and *SMN2* Genes as Main Contributors to SMA Phenotype

Two genes, *SMN1* and *SMN2*, determine the pathogenesis of SMA in humans, *SMN1* being the “causative” gene and *SMN2* the best-studied modifier. Both genes are located on the same chromosome, 5q13; the *SMN1* gene is also referred to as a telomeric SMN (*SMNt*), whereas *SMN2* is also known as a centromeric SMN (*SMNc*), reflecting their chromosomal localization. The *SMN2* gene is exclusive to Homo Sapiens, as it emerged as a result of the duplication of the *SMN1* gene that took place about 5 million years ago [16] and still shares a >99% sequence similarity with *SMN1.* There is a CNP of *SMN1* and *SMN2*; in particular, an individual might have between 0 and 8 copies of the *SMN2* gene [17]. The copy number of *SMN1* varies from 0 to (at least) 4 [18]. The possibility of a higher copy number is not excluded, although there are no studies reporting that yet. Both *SMN1* and *SMN2* genes are ~28 kb long and have 8 exons, namely exons 1, 2A, 2B, 3, 4, 5, 6 (and, rarely, 6B [19]), 7, and 8. Exon 7 is the last coding exon of these genes, and exon 8 mostly represents the 3′ untranslated region. The sequences of *SMN1* and *SMN2* differ in 11 bp; there are a few single nucleotide sequence variations distinguishing *SMN2* from *SMN1*, the only functional one amongst them being a transition NM_000344.3:c.840C>T at position 6 in exon 7 [20]. This transition disrupts a splicing enhancer sequence and subsequently leads to substantial loss of exon 7 at the stage of pre-mRNA splicing [21]. Notably, the rate of inclusion or exclusion of exon 7 is determined by several epigenetic factors, such as chromatin organization and histone marks, etc. [22].

*SMN1* encodes a functional full-length SMN protein (SMN-FL) composed of 294 amino acids, ~38 kDa; its structure has been comprehensively described [23]. The main product of *SMN2* gene expression is a SMNDelta7, a truncated form of the SMN-FL protein lacking exon 7. This form is unstable and rapidly degraded, supposedly because skipping exon 7 creates a degradation signal (degron) at C-terminal 15 amino acids of SMNDelta7; remarkably, the S270A substitution disrupts this degron and increases the stability of the SMNDelta7 protein [24]. However, in any form (stable or not), SMNDelta7 cannot completely compensate for SMN-FL loss, as they are not functionally equal. Still, some marginal amounts of processed *SMN2* mRNAs might retain exon 7 therefore, even cells without the *SMN1* gene can produce low levels of SMN-FL. The “extra copies’’ of the *SMN2* gene are supposedly not epigenetically silenced; thus, in theory, the more copies of the *SMN2* gene are present in the genome of a particular individual, the higher are the levels of SMN-FL translated from *SMN2* mRNA in all types of cells of a particular individual (which is yet to be tested). Homozygous deletions involving exon 7 of SMN2 are not pathogenic [25]. Other, shorter isoforms of the SMN protein also exist (with known and unknown functions), namely, axonal SMN (a-SMN) [26], SMN6B [19], and SMNΔ5 [27]. Their localization in the cell and patterns of expression in different tissues, both among healthy individuals and in individuals with different types of SMA, are yet to be characterized.

*SMN1* and *SMN2* genes are mapped close to each other in a highly complex locus of a 500 kb long inverted segmental duplication, which is unique to humans [28]. The standalone quality of chromosome 5 is the presence of multiple intrachromosomal duplications with a high degree of sequence identity. The region on 5q13.3, where *SMN1* and *SMN2* are located, is one of the most duplicated on this chromosome, enriched in repetitive sequences, comprising pseudogenes, and prone to unequal rearrangements and gene conversion. As discussed above, this results in a CNP of *SMN1*, *SMN2*, and other genes and pseudogenes in this region [29]. *SMN1* (and *SMN2*, given their almost identical sequences) has a very high density of non-coding transposable elements, especially Alu repeat elements, compared to the majority of the genes in the genome [30,31], which makes it prone to recombination events; for example, the most common partial deletions of *SMN1* are flanked by Alu repeat elements [32]. The question remains whether *SMN2* gene copies of the same patient are commonly identical or have some degree of sequence polymorphism. The possibility exists that “extra copies” of *SMN2* in some individuals might be epigenetically silenced (via DNA methylation or via post-transcriptional modification of histones), whereas in some individuals with the same copy number of *SMN2*, all copies might be transcriptionally active.

The aforementioned predominant causes of SMA, accounting to ~94–96% of all cases, are large deletions of the *SMN1* gene (which can also involve surrounding genes, for example, *NAIP*, *SERF1A*, and *GTF2H2A* [33,34], in patients with severe forms of SMA), or conversion of the *SMN1* gene to *SMN2.* The remaining cases of SMA are caused by the presence of loss-of-function pathogenic nucleotide sequence variants (PSVs) within the *SMN1* gene in a compound heterozygous state with the aforementioned massive deletions; such PSVs include small deletions, insertions, missense or nonsense mutations, mutations affecting splicing, etc. [31,35,36]. Very rarely, among ~5% of cases, SMA is caused by homozygous loss-of-function PSVs in *SMN1* (examples of such PSVs found in patients with SMA are summarized in [37]). Hitherto, in the *SMN1* gene, more than 80 PSVs have been identified [38].

The pan-ethnic incidence of SMA is estimated to be ~1:11 000 in live births globally [39]. The carrier frequency for SMA is different in different populations, and racial and ethnic groups, and ranges between 1:25 and 1:50 [39]. Of them, ~94% of carriers have one functional copy of the *SMN1* gene and deletion of another (phenotype 1 + 0), ~2% carry one functional copy of the *SMN1* gene and one allele of *SMN1* with loss-of-function PSV, and the remaining carriers are so-called silent carriers with two copies of the functional *SMN1* gene on one chromosome and its deletion on another (phenotype 2 + 0) [40]. The possibility of a 3 + 0 phenotype existence is also not excluded [41]. Moreover, multiple copies of the SMN1 gene are more frequent among African Americans compared to many other ethnic groups (27% vs. 3.3–8.1%) [42]. As the copy number >2 of *SMN1* per allele is highest among individuals of African descent, 2 + 0 carrier phenotype must be relatively common in this group. Alarmingly, the presence of 2 + 0 phenotype often results in a false-negative test for SMA carriers.

## 3. Biology of SMA: Cells and Tissues Affected, Multiple Functions of SMN Protein

The prominent clinical feature of SMA is dysfunction and degeneration of motor neurons in the anterior horns of the spinal cord (alpha motoneurons). Motoneurons are neuronal cells of the central nervous system (CNS) which innervate muscles, hence SMA is accompanied by atrophy of the proximal muscles and impaired respiratory function. Although in the case of SMA, the most impacted cells are alpha motor neurons, it might be considered a systemic disease [43]. Notably, according to the data from animal models of SMA, restoration of SMN in neurons alone is not enough to completely mitigate all the systemic symptoms of SMA [44]. In severe forms of SMA, when the SMN protein level is not higher than 20%, the cardiovascular system, pancreas, liver, brain, immune cells, and other types of cells, organs, and tissues are affected as well [43,45]. This is not surprising given the fact that SMN is ubiquitously expressed throughout the body, interacts with hundreds of proteins (comprehensively reviewed in [46]), and plays a role in many fundamental “housekeeping” cellular processes. To name but a few, it participates in the storage and maturation of ribonucleoprotein (RNP) complexes in nuclear Gems [47], supposedly in a de novo formation of membraneless organelles, such as Cajal nuclear bodies and stress granules [48], DNA repair and prevention of DNA double strand breaks formation [49], resolution of R-loops [49], regulation of translation (reviewed in [50]), pre-mRNA splicing [51], recognition of particular histone modification marks (such as H3K79me1) [52], translation of selenoproteins [53], mitochondrial metabolic maturation during myogenesis [54], preventing damage of ribosomal DNA [55] and many others.

Thus, a multitude of pathways are affected by the loss of SMN protein, and it is rather hard to fathom why it is a motor neuron function that suffers most in case of SMN deficiency.

However, there are several neural tissue-specific functions of SMN and features of *SMN1* gene regulation, which might explain the remarkably exclusive sensitivity of alpha motor neurons to its loss. It is known that *SMN1* gene expression can be regulated by neuron-specific pathways (stimulated by the activation of glutamate NMDA receptors) [56]. Furthermore, SMN protein participates in the local axonal translation in neurons [57] and regulates neurites outgrowth [58]. SMN is found in a complex with ribosomes, where it predominantly regulates translation of the subset of proteins involved in neurogenesis, hence its absence is exclusively detrimental for neurogenesis [59]. Several genes, whose products are important for neurons functioning, are differentially methylated in SMA, including *PAX6*, encoding transcriptional factor, which is critical for neuronal maturation; *HB9,* encoding motoneuron-specific transcriptional factor; and *CHAT*, encoding choline acetyltransferase [60]. One of the SMN isoforms prominently expressed in motor neurons, a-SMN, stimulates axonogenesis [26]. Furthermore, increased damage to ribosomal DNA (rDNA) accompanied by excessive R-loop formation and resulting in impaired ribosome biogenesis and translation has been reported in SMA. This might be impactful for motoneurons, not only because rDNA region instability is common in many neurodegenerative disorders (i.e., neuronal cells might be more vulnerable to it), but also because level of DDX21 protein, which is needed for R-loops resolving at rDNA, is a motor neurons exclusively significantly reduced in SMA [55]. The link between the rDNA (and nucleolus formed by it) and SMN and neurodegeneration is reinforced by the finding that SMN is necessary for the restoration of nucleolar organization after genotoxic stress [61]. Finally, SMN is involved in the processes of synaptic homeostasis at neuro-muscular junctions, and it has been shown that the endocytic pathways critical for normal function of neuro-muscular signal transmission are impaired in SMA [62]. In turn, restoration of secretory vesicles tethering in mice with *SMN1* deficiency was sufficient to reverse the SMA phenotype to normal [63]. Of course, the impact of glial and muscle cells’ dysfunction in SMA should not be ignored either, but still, the primary role in its pathogenesis is played by motor neurons.

Furthermore, any aberrant alterations of the SMN protein beyond physiological levels, either decrease or increase, are linked to various pathological features. For example, duplications of the *SMN1* gene, supposedly resulting in higher levels of SMN, are associated with sporadic amyotrophic lateral sclerosis [64]. In wild-type mice, long-term SMN overexpression surprisingly leads to neuroinflammation, neurodegeneration, and progressive SMA-like sensorimotor deficits through the toxic gain-of-function mechanisms [65]. These works demonstrate the importance of fine-tuning SMN levels for the normal functioning of cells and tissues.

SMN is indispensable at the early stages of development, in particular at the stage of neuromuscular junction maturation; indeed, the loss of SMN resulted in SMA-like symptoms in mice, whereas in adult animals it did not lead to SMA-mimicking phenotypes [66]. However, it has been suggested that the restoration of SMN-FL to normal levels in patients with SMA, even at the earliest stages of postnatal development, might not be sufficient to mitigate the whole spectrum of neurodevelopmental abnormalities associated with the disease [67]. Thus, the identification of novel targets for complementary therapies is needed.

## 4. SMA Therapy

The three currently available FDA-approved therapies for SMA are (1) the gene therapy Onasemnogene abeparvovec (sold under brand name Zolgensma^®^) that delivers to the cells functional *SMN1* gene via the adeno associated virus vector; (2) the antisense oligonucleotide (ASO) Nusinersen (sold under brand name Spinraza^®^) that, via displacing splicing repressors hnRNPA1/A2 from their binding site, increases the levels of mRNA with exon 7 produced from *SMN2*; and (3) the small molecule *SMN2* splicing modifier Risdiplam (sold under brand name Evrysdi^®^). Despite the recent success with development of SMN-specific SMA treatments, there is a clear need for novel and complementary therapies [68,69]. Moreover, some patients poorly respond to the existing therapy, and the molecular/genetic (or other) cause of such a phenomenon is yet to be elucidated.

Additional, SMN-independent curative approaches to SMA have been proposed, namely, inhibition of myostatin, negative regulator of muscle growth by Apitegromab, restoration of mitochondrial function by Edaravone levetiracetam, reduction of excitotoxicity in microenvironment by Riluzole/gabapentin, activation of fast troponin to increase muscle force by Reldesemtiv, inhibition of Acetylcholinesterase to improve function of neuro-muscular junctions by Pyridostigmine, and others (summarized in [69]). Overall, the potential therapy strategies for SMA, apart from the ones which are already being used in the clinic, might include (1) upregulating expression of the *SMN2* gene [70,71,72] (2) stabilizing *SMN2* transcript [73] (3) aiding the subcellular transport of *SMN2* mRNA, (4) increasing its translational efficiency [74], (5) diminishing degradation of the SMN protein [75,76] (6) targeting interaction partners of SMN, (7) targeting downstream effectors of SMN actions (for example, proteins involved in the vesicle formation at neuro-muscular junctions, etc.), and perhaps others, aimed at improving functions of organs and tissues affected by SMA and compensating for its loss. Thus, “natural” (i.e., genetically or epigenetically determined) modifiers of the SMA phenotype might be found amongst proteins involved in the regulation of all the aforementioned processes.

## 5. Established and Emerging Modifiers of SMA

The search for SMA modifiers started almost immediately after *SMN1* was established as a causative gene for this disease [77]. In this subsection of the review, we focus only on the data obtained in patient studies, describing any observations in cell lines and animal models in the next subchapter (proposed and potential DMs of SMA).

As mentioned before, of all known DMs of SMA, the most well-studied is the *SMN2* gene and its copy number, but other modifiers also exist. Apart from its copy number, *SMN2* can affect SMA via other mechanisms. For example, certain genetic features of the *SMN2* gene might affect its mRNA splicing, such as nucleotide substitution ***A****-44G* (rs212216) within intron 6 that decreases binding of a splicing repressor and is a modifier of SMA severity, associated with milder forms of the disease [78,79]. Sequence variants (SVs) *A-549G* (rs564142907) and *C-1897T* (rs1381625877) in intron 6 of SMN2 are also modifiers of SMA, in particular, they are found to be significantly associated with mild exception patients [80]. Furthermore, it has been shown that *c.859G>C* in exon 7 of *SMN2* is associated with a milder SMA phenotype, perhaps because it causes ~20% elevation of the full-length SMN RNA expression from the *SMN2* gene [81,82]. A recent study has identified two haplotypes associated with the *c.859G>C* modifier variant, so-called Smn2-859C.1 and Smn2-859C.2 haplotypes [83]. The authors of the work also identified novel variant *c.154-1141G>A*, which was detected in all patients with Smn2-859C.1 (but not Smn2-859C.2) haplotype, and was not detected in patients without *c.859G>C* modifier variant.

As for epigenetic modifications, *SMN2* can be silenced via CpG DNA methylation, and methylation at the positions −290 and −296 upstream the translation start of the *SMN2* gene is associated with the severity of SMA [84]. Predictably, methylation causes a reduction in SMN2 transcription, and reduced methylation at these sites is associated with less severe clinical features of SMA. There are also significant differences in patterns and levels of CpG methylation close to the genes *CHML, ARHGAP22, CYTSB, CDK2AP1*, and *SLC23A2* (some of them are known to be regulators of Rab and Rho GTPases) between SMA patients and healthy controls [85]. However, their contribution to SMA phenotype is yet to be assessed.

Large deletions involving genes from the same locus where *SMN1* is located, for example, *NAIP* and *SERF1A* (encoding NLR family apoptosis inhibitory protein, and Small EDRK-rich factor 1, respectively) [33,34,86] are observed in patients with severe forms of SMA, but not in mild forms. Thus, given that *SMN1* is defective in all aforementioned scenarios, the only difference being presence or absence of *NAIP* and *SERF1A* deletions, these genes are potential DMs of SMA. In line with this, the role of *NAIP* and *SERF1A* genes as SMA modifiers has been confirmed in several studies and comprehensively described in numerous previous reviews. Just one of the many examples, the study performed in 99 participants in Serbia have demonstrated that copy numbers of *NAIP* and *SERF1A* genes are modifiers of SMA. In particular, there was an inverse correlation observed between copy numbers (CNs) of *NAIP*, *SERF1A*, and *SMN2*, and the type of SMA; joint effects of the *SMN2* and *SERF1A* genes as modifiers of SMA have also been demonstrated [87]. It can be speculated that in the context of SMA, the disease modifying role of the protein encoded by *SERF1A* is due to its involvement in regulation of the protein aggregation in neurons [87]. Furthermore, proteins from the SERF family also bind RNA and localize in RNA-rich nucleolus, whereas a link between the nucleolus and SMA pathogenesis has been established [55]. Simplistically, the more copies of the gene are present in the genome, the higher is the level of the corresponding protein, and hence the more prominent its modifying effect is. In a cohort of 186 Iranian patients with SMA, it has been shown that individuals with deficient *SMN1*, 2 copies of *SMN2*, and 0 copies of *NAIP* were more likely to have SMA type 1 [88]. In the study, which included 30 patients with type 1and 35 with type 2 SMA, deletion of the *NAIP* gene was found to be associated with the severity of SMA, in particular homozygous deletion of exons 7 and 8 of *SMN2* and the absence of *NAIP* exon 5 was common amongst patients with type 1 SMA, whereas most type 2 patients had *SMN1* homozygous deletions of exons 7 and 8, but no deletions in the *NAIP* gene [89]. Notably, DMs can influence not only the severity of disease, but also the age of its onset. For example, an association of an earlier age of SMA onset, with combined genotypes of *SMN1*-*SMN2*-*NAIP*, with lower CNs has been reported. The same genotype was associated with higher and earlier mortality in SMA patients [90]. The CNs of *NAIP* also correlated with the age of disease onset in the study by Savad et al. [88]. This might be explained by the anti-apoptotic and neuroprotective role of the NLR family Apoptosis Inhibitory Protein encoded by the *NAIP* gene.

Apart from its bona fide modifier, *SMN2*, and other genes located in close proximity to *SMN1,* one of the first discovered modifiers of SMA localized on another chromosome is gene *PLS3*, encoding Plastin 3 (PLS3, also known as T-plastin). The role of Plastin 3 in the modulation of SMA severity was reported in the groundbreaking article published in *Science* in 2008, based on the transcriptome study of the aforementioned discordant families [91]. Apparently, Plastin 3 is capable of preventing the development of SMA in individuals with defects of *SMN1* and 3–4 copies of *SMN2* (reviewed in [92]). *PLS3* is an X-linked gene, thus, its SMA-protecting role is perhaps restricted to a subset of females. There is an intra-individual variability of Plastin 3 levels; in healthy individuals Plastin 3 is expressed predominantly in solid tissues [93], but can also be detected in blood cells in some individuals. It plays multiple roles in the cell; it is a Ca^2+^-dependent F-actin binding/bundling protein regulating F-actin dynamics and G/F-actin ratio [94]; it is also involved in the processes of cell migration and cell–cell contact (reviewed in [92]), and counteracts poor axonal connectivity via delayed axon pruning and improved neuromuscular junction functionality [95]. Furthermore, Plastin 3 is involved in endocytosis and its elevated levels mitigate defects of endocytosis in SMA [96]. Next, the impaired differentiation of primary motor neurons is caused, in part, by aberrant signaling by Brain-derived neurotrophic factor (BDNF). The SMN-deficiency is accompanied by a reduction in BDNF-mediated phosphorylation of tyrosine receptor kinase B receptor (TrkB), the high affinity receptor for BDNF, and its diminished restoration after BDNF stimulation [97,98]. Recently, it has been shown that Plastin 3 governs the F-actin–mediated cycle of membrane translocation and recycling of TrkB; thus, elevated levels of Plastin 3 “rescue” BDNF-TrkB signaling. It also facilitates the formation of the “cluster-like” active zone-associated voltage-gated calcium channel Cav2.2 [99]. As it has been demonstrated by Oprea et al., in families with discordant SMA, females without signs of SMA but with deletions within *SMN1* might have significantly higher levels of Plastin 3 compared to their relatives with SMA and the same defects of *SMN1* [91]. Multiple studies elucidating the role of Plastin 3 in SMA have followed since then, with contradictory results. For example, some studies also demonstrated the protective role of Plastin 3 in SMA, whereas others have not confirmed it [100,101,102]. Also, it has been shown that *PLS3* might tissue-specifically escape X chromosome inactivation, and such inactivation is epigenetically determined by the epigenetic transcriptional regulator chromodomain helicase DNA binding protein 4 (CHD4) and the macrosatellite DXZ4, which is essential for X-inactivation. In particular, *PLS3* is located ~500 kb downstream of *DXZ4* which has a high degree of copy number variability, and a significant linear correlation between number of DXZ4 copies and levels of *PLS3* mRNA was observed in females [103]. Thus, it is suggested that the DXZ4 copy number might be used as a proxy to estimate *PLS3* expression, which is a known DM of SMA. Currently, there are no Plastin 3-targeting complementary therapies for SMA, perhaps because any significant changes in its levels, either decrease or increase, are linked to a range of pathologies. For example, elevated levels of Plastin 3 are observed in various cancers and might be involved in epithelial-to-mesenchymal transition, thus potentially contributing to metastases ([104] and others). Notably, recent independent studies showed no evidence of the existence of SMA modifying sequence variants in *PLS3* at the population level [80,105].

There are also several other known DMs, including those interacting with Plastin 3 and F-actin. For example, Coronin C, encoded by the *CORO1C* gene, is another protein binding F-actin. It also binds Plastin 3 in a Ca^2+^-dependent manner [96] and is proposed to be an SMA modifier, based on its ability to rescue impaired endocytosis in human cells with SMN deficiency [96].

Neuritin 1, encoded by gene *NRN1*, has been proposed to be an SMA DM, based on its elevated expression in one of discordant siblings in a family with SMA [102].

The neuronal calcium sensor Neurocalcin delta encoded by gene *NCALD* is also a modifier of SMA. It is a Ca^2+^-dependent negative regulator of endocytosis; thus, its downregulation or loss leads to the restoration of the endocytosis in SMA models [106]. Based on the study in five asymptomatic individuals with deletions within the *SMN1* gene and only four copies of the *SMN2* gene, Reissland et al. have demonstrated that the suppression of Neurocalcin delta is a protective modifier of SMA [106]. Not surprisingly, treatment with *NCALD* antisense oligonucleotides leads to protective effects in the in vitro SMA model utilizing human motor neurons derived from induced pluripotent stem cells (iPSC) [107].

Zinc finger protein ZPR1 encoded by gene *ZPR1* is a proposed emerging modifier of SMA [108]. It interacts with SMN protein, as it is important for its functions and subcellular localization [109], and this interaction is obviously disrupted in SMA [110]. It is also a regulator of several *Hox* genes, which encode proteins critical for the function of phrenic nerve motor neurons and, hence, respiration [111]. It has been demonstrated recently that ZPR1 mitigates symptoms of SMA via preventing accumulation of R-loops and enhancing *SMN2* gene expression [72].

In a case of a SMA discordant family, nucleotide variants in the gene *TLL2* encoding an astacin-like zinc-dependent metalloprotease were found in one male patient, suggesting that they are likely to mitigate the severity of SMA [112]. This is not surprising, given that *TLL2* encodes an activator of myostatin, and myostatin negatively regulates skeletal muscle growth. As mentioned before, the inhibition of myostatin by Apitegromab has been proposed as a complementary SMA therapy.

## 6. Proposed and Potential DMs of SMA

Here, we describe potential DMs of SMA, based on in vitro cell studies and animal models, yet to be validated in patient studies. A genome-wide high-throughput RNA interference screening performed in vitro in human cells has identified several genes that encode proteins capable of modulating levels of SMN, and, hence, are potential modifiers of SMA severity. In particular, it has been found that Neurl2 E3 ligase, in cooperation with Mib1, ubiquitinates SMN, thus promoting its degradation [113]; therefore, a decrease in their levels might supposedly lead to less severe SMA. The screening revealed several potential splicing modifiers of *SMN2* mRNA, namely Heterogeneous nuclear ribonucleoproteins A2/B1, U2 small nuclear RNA auxiliary factor 1, U2 small nuclear RNA auxiliary factor 2, Poly(U) binding splicing factor 60, and Serine and arginine rich splicing factor 3 (encoded by genes *HNRNPA2B1*, *U2AF1*, *U2AF2*, *PUF60*, *SRSF3*, respectively). Furthermore, modifiers of SMN levels were found among the components of the TRanscription-EXport multiprotein complex (the major functions of this complex are coordination of transcription with mRNA transport [114], in particular THO complex subunit 1, THO complex subunit 4, nuclear transcription factor X-box binding 1, cleavage, and polyadenylation specific factor 6 (encoded by genes *THOC1*, *THOC4*, *NFX1*, and *CPSF6*, respectively)). Finally, WD repeat domain 33, cleavage and polyadenylation specific factor 1, and THO complex subunit 3 (encoded by *WDR33*, *CPSF1*, and *THOC3* genes) were found to affect SMN2 mRNA trafficking. Knocking down either of these genes resulted in an increase in SMN2 mRNA cytoplasmic localization and elevated SMN protein levels. Additionally, the knockdown of WDR33 slightly increased levels of *SMN2* pre-mRNA [113]. This study also confirmed some of the results from a transcriptome-mining study by Auslander at al., in which the authors applied GENetic moDULators identiFication (GENDULF) computational method to identify DMs of SMA by comparing transcriptomes from the specimens obtained from healthy and diseased spinal cord and muscle [115]. The top predicted DM was a known component of spliceosome, U2 small nuclear RNA auxiliary factor 2 (study by McCormack at all also confirmed its potential role as DM of SMA). Validity of several hits from this computational screen have been assessed in in vitro experiments using human cells. In line with computational predictions, in patient-derived fibroblasts, the knockdown of *U2AF1* was accompanied by elevated SMN protein levels. The next logical step would be to validate all hits from this screen in patients with different phenotypes of SMA.

Patient-specific features of alternative splicing (AS) of nascent *SMN2* RNA can also be DM in SMA. AS is regulated by many factors. They include, to name but a few, nucleotide sequence variants, ability of splicing regulators (silencers or repressors) to recognize and bind corresponding sites, levels of such regulators, and rate of transcriptional elongation by RNA polymerase II (RNA pol II), i.e., number of nucleotides added to nascent RNA per unit of time. Recently, it has been shown that the inclusion of exon 7 during the splicing of nascent *SMN2* mRNA is decreased by a slow transcriptional elongation [22]. Thus, factors which can slow RNA pol II (silencing epigenetic marks at *SMN2* locus, factors affecting chromatin occupancy at this locus, or any alterations within the RNA pol II itself, or within any RNA pol II-associated regulatory factors) would perhaps favor production of SMN2del7 [22].

There is a tendency for the genes, mostly expressed in neurons (i.e., involved in neuronal development, formation, and function of synapses), to be among the longest genes in the genome; hence, it is expected that their transcription and splicing might be particularly vulnerable to “slow” RNA pol II [116]. The RNA pol II speed varies in different types of cells and between genes [117], and can be affected by many factors. Notably, SMN directly interacts with RNA pol II [118], and the abrogation of such interaction results in SMA-like phenotypes [119]. Whether SMNdelta7 is also a member of RNA pol II interactome is not known. The way the rate of transcription is affected by the presence or absence of SMN in a complex with RNA pol II is also enigmatic. If the absence of SMN-FL (or excess of SMNdelta7) in this complex causes a decrease in rates of RNA pol II-driven transcription, which in turn leads to the shift towards production of SMN-FL over SMNdelta7 from the SMN2 gene, this will create a self-reinforcing “vicious regulatory cycle” (the slower the transcription is, the less of SMN-FL is produced, and the less of SMN-FL is produced, the slower transcription is). Moreover, the possibility exists that at an early postnatal stage, the overall transcription increases in motor neurons addressing the needs of the developing nervous system, which, if not compensated by increased levels of RNA pol II, results in slower transcription rates. All of this could be plausible explanations for the observed sensitivity of motor neurons to defects of the *SMN1* gene. Indeed, if transcriptional elongation is slow in particular cells or at the genes predominantly expressed in this particular type of cells, this would result in decreased levels of functional SMN-FL protein being translated from *SMN2*-derived mRNA exclusively in these cells. The levels of SMN-FL and SMNdelta7 protein and the corresponding mRNA (processed and pre-processed) in different cells and tissues, in healthy individuals and in individuals affected by SMA, are still largely undetermined in humans for obvious ethical issues. On the other hand, determining these levels in different types of cells generated from SMA donor-derived induced pluripotent stem cells (iPSCs), including those with different copy numbers of *SMN2*, might not be the best approach, as it is a rather artificial system affected by clonal selection bias, the absence of a tissue microenvironment, and other issues; all of this might lead to inaccurate and even erroneous findings [120]. Thus, it remains to be elucidated in further studies.

Remarkably, in a recent study by Kim et al., the pathogenic SMA phenotype was almost completely reversed back to normal in mice with a lack of a functional *SMN1* gene but the presence of single nucleotide variant in the gene *Hspa8*, leading to G470R amino-acid substitution in the heat shock protein 70 (HSPA8) protein encoded by this gene [63]. Such a striking impact on SMA phenotype, much stronger compared to the impact of other known modifiers, apart from *SMN2*, gave this variant a name of “shocking modifier of SMA” [121]. HSPA8 is a chaperone that interacts with SMN and has several neuron-exclusive roles, such as the maintenance of neuronal dendritic proteostasis [122] (notably, the connection of the SMN complex with the proteostasis network has been proposed recently [123]). The main role of HSPA8 in synapsis is as a chaperone participating in the formation of the SNARE, a key protein complex needed for secretory vesicles tethering. As it was demonstrated by Kim et al., the Hspa8 G470R variant both promoted assembly of the SNARE (which was otherwise disrupted in SMA) and slightly increased SMN protein levels [63]. The functionally similar variants in the human gene encoding heat shock protein 70 remain to be identified, and the possibility to pharmacologically target SNARE complex formation in SMA is yet to be explored. Overall, these findings reinforce the view that SMN is not the only target in SMA, and that despite multiple roles of SMN and its involvement in a plethora of cellular pathways, it is dispensable and replaceable in most of them, except for motor neuron-specific, such as, signal transduction at neuro-muscular junctions via secretory vesicle signaling.

Non-coding RNAs (ncRNAs), including long non-coding RNAs (lncRNAs) and microRNAs (miRNA), orchestrate transcriptional and post-transcriptional gene expression regulation and determine gene expression changes at critical steps of cell differentiation, post-natal tissue development, and growth both in health and disease (reviewed in [124,125]). They might have tissue-specific patterns of expression; their levels vary depending on the development stage and changes in response to particular stimuli. A group of miRNAs, named motomiRs, play a vital role in regulating motor neuron functions [126]. Thus, it is not surprising that non-coding RNAs can be involved in motor neuron development and diseases [127,128], including SMA. In particular, two teams independently have identified lncRNA SMN-antisense 1 (SMN-AS1) that can repress expression of the *SMN2* gene [70,71]. However, comparative transcriptomic studies of ncRNAs in patients with different clinical severities of SMA and identical *SMN1* sequence loss have not been performed yet.

Finally, modifiers not only of SMA manifestation and progression but also of the therapy response must exist; however, we left them beyond the scope of this concise review.

## 7. Conclusions

SMA is a devastating neurodegenerative condition, which poses a significant burden on the healthcare system [129], especially given the high cost of the current therapy for the most severe forms of the disease. Despite being a monogenic disease, SMA is a condition with a “continuum of clinical severity”, modulated by several DMs. In this narrative review we provided an overview of existing, emerging, and proposed DMs of SMA [Supplementary Table S1].

In the context of SMA, of all the DMs discussed above, only *SMN2* and *NAIP* CNs, as well as levels of Plastin 3 protein, have been comprehensively investigated by many independent teams, whereas others are much less studied and their role as DMs is still under question. Thus, more robust studies in different populations (given potential race- or ethnic-specific features of any DMs), including multiple family studies and studies at population level, are needed to assess the impact of the proposed DMs and their combinations on the severity of SMA. Further actionable steps should include the assessment of the proposed potential DMs in large-scale patient studies, including multiple family studies, as well as the side-by-side validation of the clinical utility of all previously established DMs, including the controversial and less-studied ones, and the search for novel DMs among regulatory miRNAs and other biomolecules previously understudied in the context of SMA.

From the point of view of feasibility of such further studies and the transferability of the study results to healthcare, perhaps priority must be given to genome-, rather than epigenome-, transcriptome-, or proteome-based investigations. This is, first and foremost, because the epigenetic marks, mRNA and protein levels of particular DMs, might be cell-specific and tissue-specific (for example, motoneuron-exclusive), whereas almost any type of DNA-containing biomaterial, including archived DNA, dried blood spots, etc., can be used for genetic analysis.

Undoubtedly, the discovery and characterization of all possible DMs can lead to the identification of novel druggable therapeutic targets (in other words, to the DMs-driven discovery of a yet unknown “Achilles heel” of the disease), aid in enhancing existing curative approaches, and allow for developing prognostic biomarkers of the disease, fine-tuned stratification of the patients, and personalization of the treatment.

## Data Availability

No new data were created.

## Supplementary Materials

The supporting information can be downloaded at: https://www.mdpi.com/article/10.3390/ijms252011210/s1.
