# Supplementary material for: In Search of Spinal Muscular Atrophy Disease Modifiers
Source: Int J Mol Sci. 2024 Oct 18;25(20):11210. doi: 10.3390/ijms252011210 (PMC11508272; doi:10.3390/ijms252011210)
Supplement: Supplementary file 1 [file ijms-25-11210-s001.zip › ijms-3232662-supplementary.pdf]

International Journal of *Molecular Sciences*

Table S1. SMA disease modifiers*.

Disease modifier	References*
**Established / identified in human studies**	
**Level of SMN protein determined by**<br>*SMN2* copy number / expression level<br>*SMN2* SVs and their combinations with SVs in other genes<br>*SMN2* promoter CpG methylation	 [7,8,87]<br>[78–83]<br>[84]
NAIP copy number	[88–90]
SERF1A copy number	[87]
**Plastin 3** expression level	[91,92,100–103]
**Coronin C** expression level	[96]
**Neuritin 1** expression level	[102]
**Neurocalcin delta** expression level	[106,107]
**Zinc finger protein ZPR1** expression level	[72,108]
TLL2 SVs	[112]
**Proposed / identified *in vitro* or in animal studies**	
**Level of SMN protein determined by**<br>Proteasomal degradation<br>mRNA transport<br>Splicing**	[113]<br>[113]<br>[22,115]
**Non-coding RNAs**	[70,71]
Hspa8 SVs impacting formation of SNARE complex***	[63]

* selected; **modulated by factors other than SVs in *SMN2;* *** demonstrated in animal model only.
